# Carbonyls and Aerosol Mass Generation from Vaping Nicotine Salt Solutions Using Fourth- and Third-Generation E-Cigarette Devices: Effects of Coil Resistance, Coil Age, and Coil Metal Material

**DOI:** 10.1021/acs.chemrestox.3c00172

**Published:** 2023-09-12

**Authors:** Lillian N. Tran, Elizabeth Y. Chiu, Haylee C. Hunsaker, Kuan-chen Wu, Brett A. Poulin, Amy K. Madl, Kent E. Pinkerton, Tran B. Nguyen

**Affiliations:** † Department of Environmental Toxicology, University of California, Davis, Davis, California 95616, United States; ‡ Department of Chemistry, University of California, Davis, Davis, California 95616, United States; § Center for Health and the Environment, University of California, Davis, Davis, California 95616, United States

## Abstract

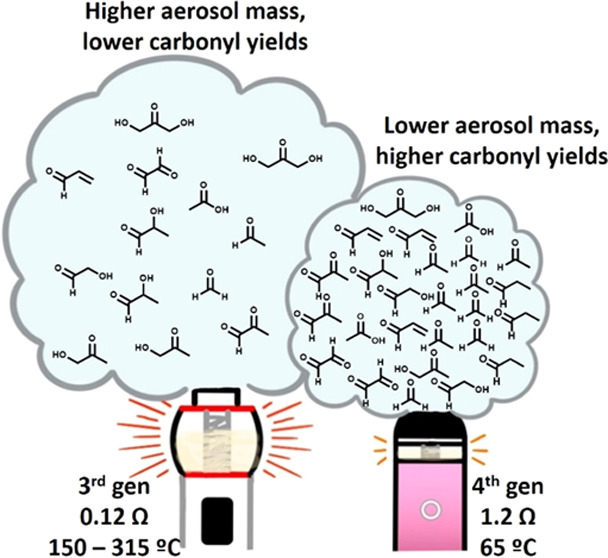

Aerosol formation and production yields from 11 carbonyls (carbonyl concentration per aerosol mass unit) were investigated (1) from a fourth-generation (4th gen) e-cigarette device at different coil resistances and coil age (0–5000 puffs) using unflavored e-liquid with 2% benzoic acid nicotine salt, (2) between a sub-ohm third-generation (3rd gen) tank mod at 0.12 Ω and a 4th gen pod at 1.2 Ω using e-liquid with nicotine salt, together with nicotine yield, and (3) from 3rd gen coils of different metals (stainless steel, kanthal, nichrome) using e-liquid with freebase nicotine. Coil resistance had an inverse relationship with coil temperature, and coil temperature was directly proportional to aerosol mass formation. Trends in carbonyl yields depended on carbonyl formation mechanisms. Carbonyls produced primarily from thermal degradation chemistry (e.g., formaldehyde, acetaldehyde, acrolein, propionaldehyde) increased per aerosol mass with higher coil resistances, despite lower coil temperature. Carbonyls produced primarily from chemistry initiated by reactive oxygen species (ROS) (e.g., hydroxyacetone, dihydroxyacetone, methylglyoxal, glycolaldehyde, lactaldehyde) showed the opposite trend. Coil age did not alter coil temperature nor aerosol mass formation but had a significant effect on carbonyl formation. Thermal carbonyls were formed optimally at 500 puffs in our study and then declined to a baseline, whereas ROS-derived carbonyls showed a slow rise to a maximum trend with coil aging. The 3rd gen versus 4th gen device comparison mirrored the trends in coil resistance. Nicotine yields per aerosol mass were consistent between 3rd and 4th gen devices. Coil material did not significantly alter aerosol formation nor carbonyl yield when adjusted for wattage. This work shows that sub-ohm coils may not necessarily produce higher carbonyl yields even when they produce more aerosol mass. Furthermore, carbonyl formation is dynamic and not generalizable during the coil’s lifetime. Finally, studies that compare data across different e-cigarette devices, coil age, and coil anatomy should account for the aerosol chemistry trends that depend on these parameters.

## 1 Introduction

Since its introduction to the U.S. market in 2007, electronic (e-) cigarette technology has continuously evolved in order to increase vaping efficiency and maintain appeal among nicotine users.^1−3^ There are multiple variables within the process of vaping an e-cigarette, including the type (the design or “generation”) of device, composition of the e-liquid (e.g., carrier solvent ratios, freebase nicotine or nicotine salt content, and flavoring chemicals), and the metal material, age, and shape of the coil.^3^ E-cigarette devices include first-generation cig-a-likes, second-generation vape pens, third-generation (3rd gen) tank-mods, and now, fourth-generation (4th gen) pods and pod-mods.^3^ The 3rd gen devices alone are manufactured in a variety of styles: different coil materials (stainless steel, nichrome, kanthal, nickel), single-mesh or multiple-mesh coil designs, cotton or ceramic wick, and different resistances that operate with power settings ranging from 10 to 140 W.^3−5^ The stainless steel and titanium coils in 3rd gen devices allow for temperature control through the device settings. 4th gen pods or pod-mods, which have been recently introduced to the market and are popular among nicotine users, are designed to vape nicotine salts. 4th gen devices are also designed for more efficient nicotine delivery and can be purchased as refillable pods or prefilled pods in a range of formulations containing various organic acid additives and nicotine concentrations.^6−11^

The increasingly diverse e-cigarette landscape provides a substantial challenge for the study of toxicant formation and aerosol generation (where the term aerosol includes both gas and particle phase emissions) from e-cigarettes. It is difficult to generalize the results of studies testing specific device conditions^11−14^ due to diverse application use. Normalizing results by the nicotine concentration in the aerosol or by the wattage of the device helps to facilitate the uniformity of enhancing study designs across multiple variables.^15,16^ However, there continues to be a need for investigations of those fundamental device parameters such as differences in device types and generations, coil metal material, coil age, and coil resistance using realistic and well-controlled e-liquid compositions to better understand and reconcile results from various studies.

Specifically, there is an uncertainty regarding whether lower coil resistance (generally leading to higher power or higher temperature) produces more of certain carbonyl compounds compared to higher-resistance devices. There is also an ambiguity in reporting the behavior of carbonyls as a single compound class. Simple aldehydes, simple ketones, and hydroxylated aldehydes and ketones all exist in e-cigarette aerosols, some of which are known carcinogens like formaldehyde and acrolein,^17,18^ and various carbonyls may be formed via different chemical mechanisms.^19^ Research data are mainly reported for carbonyls for which calibration standards are commercially available;^20^ these tend to be the carbonyls produced primarily by thermal degradation mechanisms of the e-cigarette solvents, propylene glycol (PG) and vegetable glycerin (VG). We use the term “thermal carbonyls” to refer to those formed primarily by heat-induced dehydration—acrolein, formaldehyde, acetaldehyde, and propionaldehyde.^19,21−24^ Carbonyls can also be produced by initial reaction with reactive oxygen species (ROS);^19,25,26^ many of these are less-studied in the literature in the context of e-cigarettes. We use “ROS-derived” carbonyls to refer to those that may be significantly formed via mechanisms such as hydroxyl radical H-abstraction, e.g., hydroxyacetone, dihydroxyacetone, glyoxal, glyceraldehyde, glycolaldehyde, etc. However, some carbonyls are formed via both mechanisms, and some may be formed exclusively by PG or VG or by both polyols, which further amplify the complexity in studying the formation of carbonyls. Yet further carbonyls are formed from flavorant additives.

The 3rd gen “sub-ohm” devices (0.1 Ω ≤ coil resistance < 1 Ω) have been shown to produce higher amounts of certain carbonyls when measured as μg per puff, compared to lower-powered devices with higher resistance.^22,27−32^ However, this may be due to the selection of carbonyls or the fact that a sub-ohm device generates more aerosol mass in general, compared to a high-resistance device. Aerosol mass is typically not accounted for when reporting carbonyl formation but could be important as the higher aerosol mass represents a higher nicotine concentration. Users tend to self-titrate nicotine, which may result in users of different e-cigarette generations inhaling a similar or different dosage of carbonyls. Beauval et al. normalized their carbonyl results by e-liquid consumed and found that carbonyl concentrations in a higher-powered device were neither significantly nor consistently higher than the lower-powered second-generation device.^33^ Talih reached a similar conclusion that higher-power devices do not necessarily correlate with more volatile aldehyde emissions and that device design has a stronger influence over formation of carbonyls.^34^ Pinkston and co-authors found significantly lower cell viability, higher lactate dehydrogenase levels, and higher extracellular ROS when normalized by nicotine dose from the 4th gen system (using nicotine salt) compared to the 3rd gen system (using freebase nicotine).^15^ In order to better isolate the effects of coil resistance, it is necessary to account for the aerosol mass produced and collected, nicotine mass produced, type of carbonyl produced, and the effect of coil temperature.^4,16,30,33,35,36^ These considerations should extend to whether the device regulates power in response to resistance in order to stabilize the temperature of the coil.

Saliba et al. studied the production of eight carbonyls from different metal wires (stainless steel, kanthal, nichrome); they showed that the coil material also affects the formation of carbonyls in a proxy vaping scenario.^37^ However, their study used an uncommon e-liquid (pure PG, without nicotine) and a custom setup of a coil dipped in the solvent, which is different in the size, shape, and wicking mechanism from commercial e-cigarette devices. In addition, there is also the finding that “aged” coils could produce higher levels of some carbonyls and free radicals in 3rd gen and pyrolysis devices up to 900 puffs or until the coil discolor, compared to new coils.^22,37,38^ However, there is no uniform definition of an “aged” coil in the literature, and the trends are unknown up to several thousand puffs that many vaping coils can deliver before they are disposed. These studies on coil composition and age would benefit from investigation with the newer 4th gen device due to the different contact mechanism between coil and e-liquid.

Indeed, most studies in the literature have focused on the first-, second-, third-generation or surrogate vaping devices.^4,27−29,39^ Recent studies on 4th gen devices characterized a range of potentially harmful compounds (carbon monoxide, polycyclic aromatic hydrocarbons, carbonyls, metals),^40−44^ suggesting that the higher-resistance pods and pod-mods may not necessarily offer the benefit of increased nicotine aerosolization without harmful side products. A systematic investigation of aerosol mass and normalized carbonyl formation (from both thermal and ROS-derived mechanisms) in 4th gen devices as a function of coil resistance, age, and material is needed. Furthermore, a chemical comparison between a 4th gen device and the 3rd gen sub-ohm tank has not been reported, although a recent study compared the in vitro toxicity of the aerosols produced from 3rd gen and 4th gen systems.^15^ Studies using refillable 4th gen devices in combination with a highly controlled e-liquid could help to isolate the impacts of individual device hardware components to the emissions of harmful compounds. For example, studies on prefilled flavored pods,^43,44^ for which information on ingredients is proprietary, may be subject to greater chemical complexity and inhomogeneity across brands.

Here, pertinent questions regarding current trends in e-cigarette vaping were evaluated using a well-controlled e-liquid with commercially relevant nicotine salt concentrations and composition including the following: (1) How does power and coil resistance translate into coil temperature, aerosol formation, and carbonyl formation yields in a 4th gen e-cigarette pod device? (2) Do 4th gen pod coils aged up to several thousand puffs produce different aerosol mass and carbonyl yields compared to new coils? (3) Do sub-ohm devices produce a higher aerosol mass, carbonyl yields, and nicotine yields compared to higher-resistance (lower-powered) devices? (4) Does the coil metal material affect the production of aerosols or carbonyls under typical use conditions? The data provided by this study may offer a fundamental basis for comparison across studies and aid in improved assessment of the hazards and risks of vaping from different device types and conditions.

## 2 Methods

### 2.1 E-Liquid Formulation

E-liquid formulations with freebase (FB) nicotine, used for the metal coil material study, were prepared with 6 mg/mL nicotine (0.6% FB) in 30% propylene glycol (99%, from Sigma-Aldrich) and 70% vegetable glycerin (≥99.5% from Sigma-Aldrich) by volume (i.e., 30:70 PG/VG), with relevance to commercially available e-liquids.^45,46^ Solutions were stored at 2–8 °C prior to use. E-liquid formulations with nicotine salts were prepared at 2% (w/w) or 20 mg/mL, with relevance to commercially available e-liquids (20–50 mg/mL concentration range).^46−48^ Benzoic acid (≥99.5%, from Sigma-Aldrich), was chosen as the acid for the nicotine salt (1:1 molar ratio with nicotine)^7,8^ due to its popularity in commercial formulations.^9,10,46−48^

### 2.2 Generation of Aerosol: A Fourth-Generation Device

**Figure 1:**
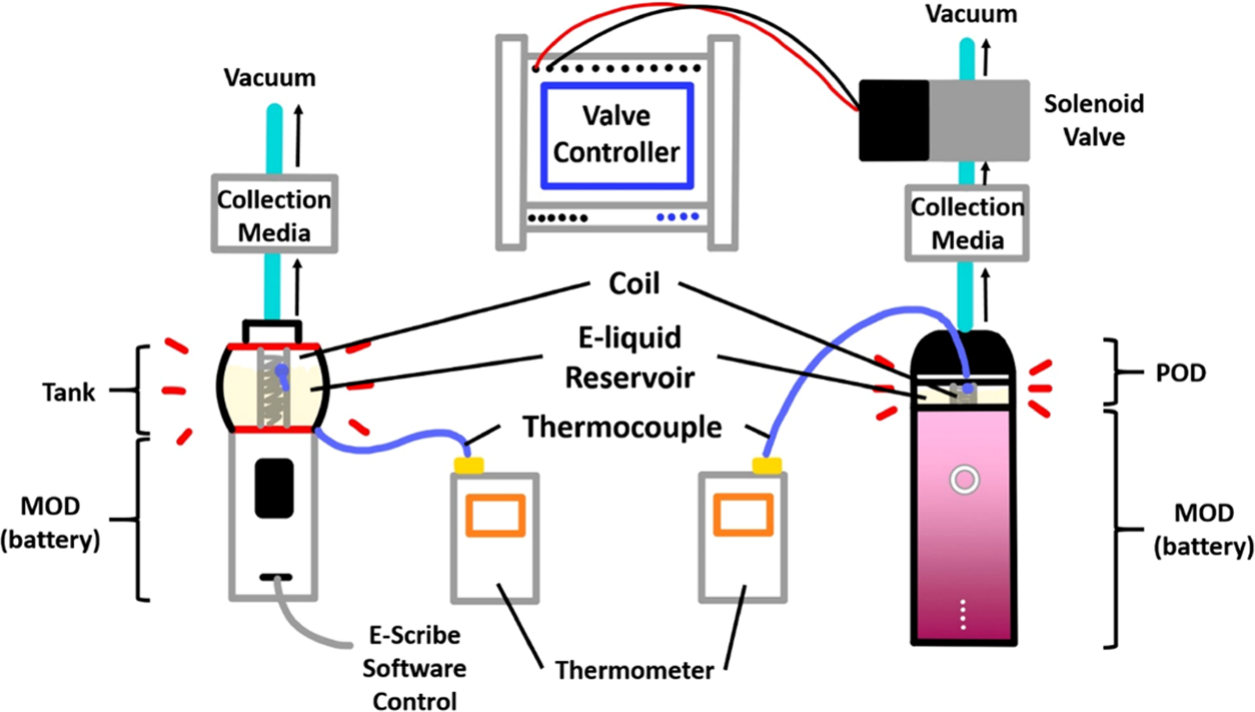
Schematic of 3rd and 4th generation vaping device control and sampling setup.

The Vaporesso XROS 2 pod device (Shenzhen Smoore Technology Limited, Shenzhen, China) was used as the 4th gen device for aerosol generation. The Vaporesso pods are refillable, allowing precise control of the e-liquid formulation. This device has a battery capacity of 1000 mAh. Three resistances were chosen for testing: 0.6, 0.8, and 1.2 Ω. Pods had built-in kanthal (FeCrAl alloy) mesh (0.6 and 0.8 Ω) or wire (1.2 Ω) coils with cotton wicking (Figure S1). Vaporesso advertised that 0.6 Ω is the best for vaping freebase nicotine and 1.2 Ω is the best for vaping nicotine salts. The device was activated with an average applied vacuum flow of 1.95 ± 0.16 L/min and a puffing regimen controlled by solenoid valves that were operated with a time relay controller (PTR4-SP, Changzhou Xuchuang Info. Tech. Co., Changzhou, China) (Figure 1). The flow rate required for activation of the 4th gen device was higher than that for the 3rd gen. We chose to keep the puff volume similar between the 4th and 3rd gen studies (Section 2.3); thus, a 2 s puff duration was used to achieve a puff volume of 65 ± 5 mL, which is also well within the typical range of puffing topography for e-cigarette users.^49^ The puff frequency was 2 puffs/min. For coil aging studies, the 1.2 Ω pod coil was chosen. We first collected carbonyls from a new unaged coil (0 puffs) and aged it up 5000 puffs. Carbonyls were collected in 50 puff intervals up to the 1000 puff age, 500 puff intervals starting at the 1000 puff age, and then to 1000 puff intervals starting from the 2000 puff age. Each set of samples was collected with a new pod to minimize coil aging effects, except when studying coil aging. The temperatures of the 4th gen coils were measured separately from the experiments, using a flexible K-type thermocouple (Frienda, Shenzhen Chenying Network Technology Co., Ltd.) in contact with the center of the pod (Figure 1), measuring the temperature continually throughout the puff. The average temperature was recorded once the temperature stabilized. The temperature output was read with a digital HH506A thermometer (Omega Engineering, Inc, Norwalk, Connecticut). 30 puffs were used for temperature measurements.

### 2.3 Generation of Aerosol: A Third-Generation Device

A Centaurus DNA 250C 200 W tank mod (Lost Vape, Shenzhen, China) was used in conjunction with the SMOK (Shenzhen IVPS Technology Co., Shenzhen, China) and FreeMax (FreeMax Technology Co., Shenzhen, China) coils (Figure 1). The Centaurus DNA tank mod may deliver a power range from 1 to 200 W. We investigated three metal materials for single-mesh coils in the 3rd gen device: stainless steel (FeCrNiMoC alloy), kanthal (FeCrAl alloy), and nichrome (NiCr alloy). Coils were chosen based on availability; stainless steel (SSF, 0.12 Ω) and kanthal (KLF, 0.14 Ω) coils were sourced from FreeMax, and kanthal (KLS, 0.3 Ω) and nichrome (NCS, 0.15 Ω) coils were sourced from SMOK. Based on the manufacturer’s label, the SSF and KLF coils operated with a power range from 20 to 70 and 40 to 70 W, respectively. The KLS coil operated with a power range from 80 to 140 W (best at 100 to 110 W) and the NCS coil operated best at 120 W. There were important differences between the two brands, namely, the anatomy of the coils and the tank reservoir design (Figure S2).

The tank mod had an adjustable airflow valve, used to create larger or smaller “clouds” or concentrate flavor.^50,51^ The aerosol mass produced can be impacted by this airflow valve; a closed valve produces less aerosol mass in comparison to a completely opened valve (Figure S3). For consistency in the sample collection, the airflow valve was kept completely open for all samples collected; thus, these data on the 3rd gen devices represented the maximum aerosol volume with the same vacuum flow rate and puff duration.

All samples were collected with new coils to avoid coil aging effects. Evolv Escribe software (Evolv LLC., Hudson, Ohio) was used to control the puff duration (3 s) and set the power supplied to the coil. The KLF, KLS, and NCS coils are operable in power mode only, whereas SSF was operated on temperature control mode. To minimize variance in the data caused by the differences in coil resistance, the power on the Escribe software was adjusted to achieve the same output temperature from each tested coil. In accordance with the CORESTA puffing protocol (3 s duration, 55 mL volume),^52^ the puff volume was controlled to 60 ± 5 mL with a 3 s puff and vacuum flow rate of 1.19 ± 0.11 L/min. The puff frequency was 2 puffs/min.

The temperature of each coil during device operation was measured with a flexible K-type thermocouple in contact with the center of the coil mesh, as done for the 4th gen device (Figure 1). In the comparison study between the 4th and 3rd gen devices, a 1.2 Ω pod was selected for vaping nicotine salts and the 0.12 Ω SS316L coil in the tank mod was selected, respectively. A SS316 coil was chosen for the comparison study for its temperature control properties.

### 2.4 Aerosol Mass Measurement by Gravimetric Analysis

E-cigarette devices that had been filled with e-liquid (6 mL for tanks, 1 mL for pods) were weighed before and after vaping to assess gravimetric mass loss of the e-liquid for a certain number of puffs. The total aerosol mass per puff was calculated as the difference in mass of the e-liquid reservoir divided by the number of puffs that were generated
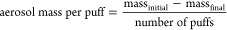
1

### 2.5 Collection and Analysis of Carbonyls and Benzoic Acid by HPLC-HRMS

The methods for the collection and analyses of carbonyls used in this work have been described previously.^19,20^ Briefly, the total aerosol was sampled through a 2,4-dinitrophenylhydrazine (DNPH) cartridge (350 mg DNPH, Supelco, Inc., Bellefonte, PA), which quantitatively (≥98.4%)^19^ derivatizes the carbonyls into hydrazones for analysis. All samples were collected in triplicate. Cartridges were extracted at ≥97% efficiency^19^ with 2 mL of acetonitrile (liquid chromatography–mass spectrometry (LC-MS) grade, Fisher Scientific Inc., Hampton, NH) prior to high-performance liquid chromatography–high-resolution mass spectrometry (HPLC-HRMS) analysis. Separation occurred on an Agilent 1100 HPLC using an Agilent Poroshell EC-C18 column (2.1 mm × 100 mm, 2.7 μm, 120 Å) coupled to a linear trap quadrupole–Orbitrap (LTQ-Orbitrap) mass spectrometer (Thermo Corp., Waltham, MA) at a mass resolving power of 30,000 *m*/Δ*m* at *m*/*z* 400.

Formaldehyde–, acetaldehyde–, acetone–, acrolein–, and propionaldehyde–DNPH hydrazones were calibrated and quantified with commercial analytical standards (AccuStandard, New Haven, CT) (Figure S4). Acetic acid and glycolaldehyde DNPH hydrazones standards were synthesized as described previously.^53^ Other carbonyls were quantified using theoretical calculations of relative sensitivity in the electrospray ionization (ESI) negative mode ionization, and ratioed to measured sensitivities of commercial standards.^19,20^ The uncertainty of the analysis is 10–20% when using analytical standards and 30–50% when using the theoretical model.^19,20^ Free benzoic acid (not derivatized by DNPH) was captured in the silica matrix of the DNPH cartridge; it was calibrated by commercial standards and quantified by HPLC-HRMS. Collection cartridges were weighed to determine the total aerosol collected. Approximately 50 puffs were collected on the cartridge for studies with the 4th gen device, such that the derivatization agent remains in excess. To keep a consistent level of aerosol mass collected, approximately 20 puffs were collected for studies with the 3rd gen device. Concentrations of each carbonyl were normalized by the amount of aerosol collected on the cartridge
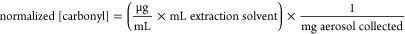
2The observed carbonyls represent approx. 90% or greater of the total DNPH-derivatized peak areas in the HPLC-HRMS analysis. Mass fractions of carbonyls (calculated mass normalized by total carbonyl mass) are tabulated in the Supporting Information.

### 2.6 Collection and Analysis of Nicotine by GC-MS

Nicotine was collected on a 47 mm Pallflex Tissuquartz air monitoring filter (Pall Corporation, Cortland, New York). The filter collection efficiency of the aerosol on average was ∼85% for both 3rd and 4th gen devices when comparing the aerosol mass collected and the total mass loss of the device (Figure. S5). Filters were extracted by sonicating with 5 mL of toluene (≥99.5%, obtained from Sigma-Aldrich). The filter extracts were diluted by 10 before analysis. Nicotine was separated on an Agilent 6890 gas chromatograph with an HP5-ms UI capillary column (30 m, 0.25 mm ID, 0.25 μm film, Agilent Technologies Inc., Santa Clara, CA) and analyzed with an Agilent 5973N mass spectrometer. The injection mode was splitless with an injection volume of 1 μL, an inlet temperature at 250 °C, purge flow at 15 mL/min for 1 min. The oven program was as follows: 70 °C for 2 min, 20 °C/min ramp to 230 °C, hold for 1 min. Quantification was performed using nicotine analytical standards (Figure S6).

### 2.7 Statistical Analysis

**Figure 2:**
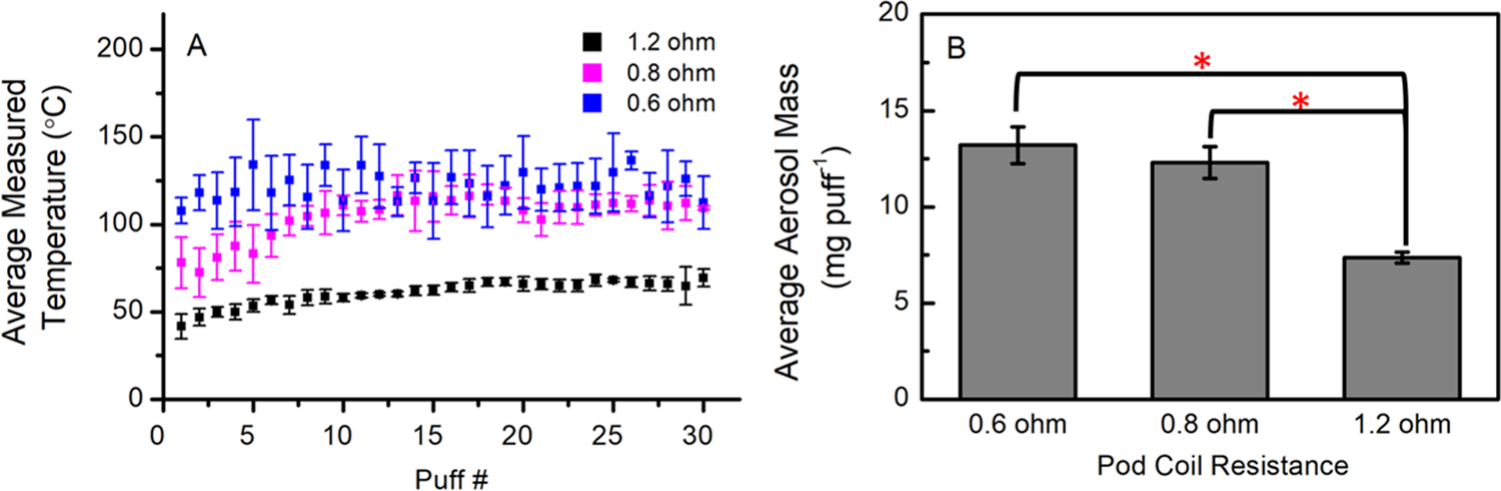
(A) Measured temperature (average ± SD) of each pod resistance in °C, and the (B) aerosol mass (mg puff^–1^) (average ± SD) generated from vaping 2% nicotine salt on different pod coil resistances. Asterisks (*) denote statistically significant differences (*p* < 0.05) in panel (B). In panel (A), the average temperatures for 0.6 and 0.8 Ω are not significantly different, but both groups are significantly different from the 1.2 Ω temperatures.

**Figure 3:**
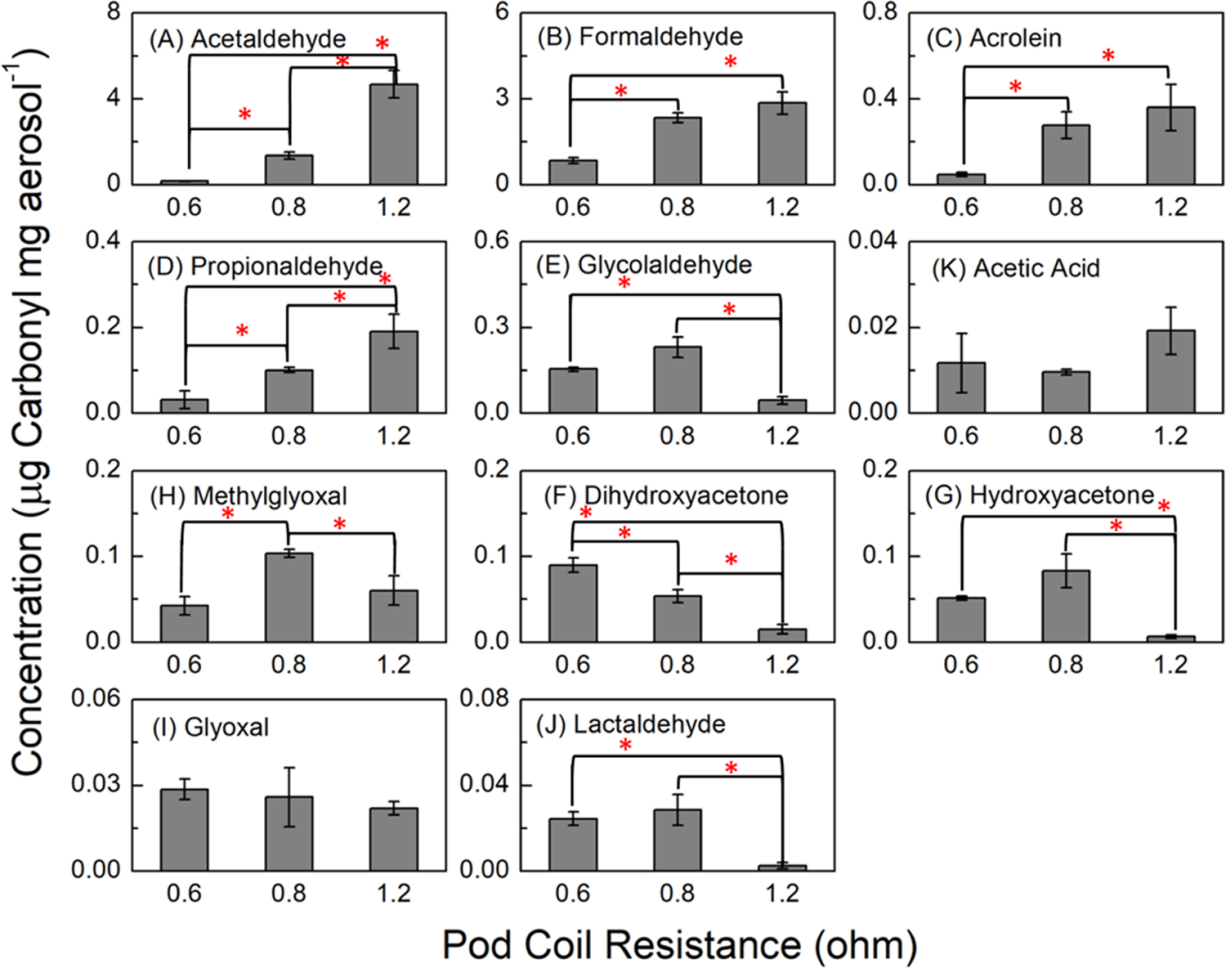
Concentration (μg carbonyl mg aerosol^–1^) of detected carbonyls in the aerosol (average ± SD) from vaping 2% nicotine salt on different pod coil resistances. Asterisks (*) denote statistically significant differences (*p* < 0.05).

**Figure 4:**
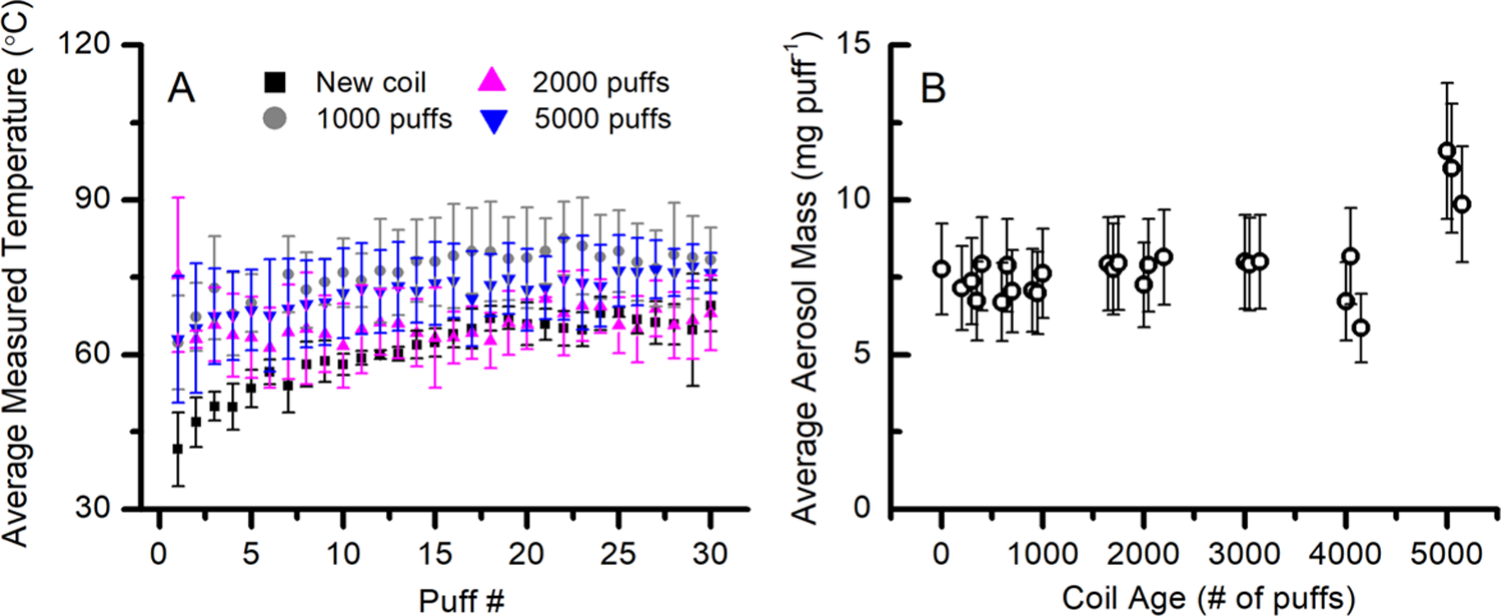
Temperature measured for a new 1.2 Ω pod coil, a 1000 puffs aged coil, 2000 puffs aged coil, and 5000 puffs aged coil, averaged over three 30 puff trials using 2% nicotine salt. Error bars indicate one standard deviation from repeated trials. In panel (A), the average temperatures for all coil ages were not significantly different from each other (*p* > 0.05). In panel 4(B), coil age did not correlate with aerosol mass.

A one-way ANOVA test (α = 0.05) was performed for the data in Figures 2A,B, 3, and 4A. If the ANOVA test showed a significant difference, the Tukey HSD test was performed to determine which pairs were significantly different from each other. A two-tailed *t*-test was performed for Figures 6A,B and 7. A linear regression was performed for Figure 4B. Carbonyl and aerosol mass measurements had sample sizes of 3, whereas temperature measurements had sample sizes of 30.

## 3 Results and Discussion

### 3.1 Effect of Pod Coil Resistance on Aerosol Mass and Carbonyl Production

Device experimental conditions discussed in this work are shown in Table 1. Figure 2A shows that the pod’s coil resistance is inversely proportional to measured coil temperature, i.e., lower-resistance coils can reach higher temperatures. Furthermore, the measured coil temperature in a 4th gen device is directly proportional to observed aerosol mass production (Figure 2A,B). This agrees with the current literature understanding that lower-resistance coils produce more aerosol mass,^16,18−28,22,47^ suggesting that the 4th gen device behaves similarly in aerosol generation as older generation devices. The data are tabulated in Table 2.

**Table 1:** Experimental Conditions for the 3rd and 4th Gen Devices Tested in This Work Using an E-Liquid Composition of 2% Nicotine Salt (1:1 Nicotine/Benzoic Acid by Mole, 20 mg/mL Nicotine)

device type	coil material	brand	coil resistance (Ω)
3rd gen (tank mod)	stainless steel (SSF)	Freemax	0.12
4th gen (pod)	kanthal	Vaporesso	1.2
4th gen (pod)	kanthal	Vaporesso	0.8
4th gen (pod)	kanthal	Vaporesso	0.6

**Table 2:** Power Output, Measured Temperature, and Aerosol Mass (Average ± SD) Generated from Vaping 2% Nicotine Salt on Different 4th Gen Pod Coil Resistances

pod coil resistance (Ω)	mfg. labeled power output (W)	measured temperature	aerosol mass (mg puff^–1^)
0.6	21	122 ± 15 °C (252 °F)	13.2 ± 1.0
0.8	16	103 ± 15 °C (217 °F)	12.3 ± 0.8
1.2	10	63 ± 8 °C (145 °F)	7.4 ± 0.3

In contrast to aerosol mass production, we observe that the mass-normalized concentration yields (μg mg aerosol^–1^) of some carbonyls (Figure 3) increase with increasing resistance, while some others exhibit different trends. The 1.2 Ω pod, which generates the lowest aerosol mass, produces the highest normalized yield of total carbonyls. A comparison of the carbonyls individually shows that some of them (glycolaldehyde, dihydroxyacetone, hydroxyacetone, and lactaldehyde, Figure 3E–J) are produced in significantly higher concentration yields from the lower-resistance pods (0.6 and 0.8 Ω). The 0.6 and 0.8 Ω pods produce very similar aerosol mass (Figure 2B). Conversely, concentration yields of acetaldehyde, formaldehyde, acrolein, and propionaldehyde (Figure 3A–D) are significantly higher from the 1.2 Ω pod compared to the lower-resistance pods. And the remaining carbonyls (acetic acid, methylglyoxal, and glyoxal, Figure 3H,I,K) are similar in concentration yields among the different resistance pods.

It is not clear why the yields of acetaldehyde, formaldehyde, acrolein, and propionaldehyde increase with increasing coil resistance (i.e., decreasing measured coil temperature). These carbonyls are identified to be formed primarily from a simple thermal degradation mechanism, instead of through reaction with a hydroxyl radical or other reactive oxygen species (ROS).^20,25^ This suggests that the higher-resistance coil in a 4th gen device may increase localized e-liquid temperature or increase catalytic degradation capacity of the coil independent of coil heating. Deconstructing the pods reveals that coils can vary in thickness, surface area, and shape to alter the resistances (Figure S1). The 1.2 Ω coil has a thin wire-like appearance as opposed to a flat mesh coil that is found in the 0.6 and 0.8 Ω pods. In a closed electrical circuit, current flows from the battery through a resistor, producing a voltage drop. In our e-cigarette systems, the current from the battery is constant regardless of the coil resistance. But the voltage drop when the current reaches the coil depends on the coil resistance.^54^ A higher resistance could produce a larger voltage drop,^54^ meaning more energy is dispersed to the surrounding e-liquid to lower the current; this is “energy dissipation” from the resistor. A lower current through the coil translates to a lower temperature at the surface of the coil (Figure 2A); however, more energy is dispersed to the surrounding wick and e-liquid when there is a larger voltage drop. This can potentially explain why more thermally generated carbonyls are produced in devices with high coil resistance (and unflavored e-liquids). Ultimately, future research studies pertaining to the physics of coil and e-liquid vaporization are needed to better understand the reasons behind the observed trends. It is not clear if the use of other e-liquids with flavorant additives would produce the same results.

For the carbonyls proposed to be produced by the ROS-initiated mechanism from Li et al.,^19^ hydroxyacetone, lactaldehyde, glycolaldehyde, and dihydroxyacetone, it is possible that the smaller surface area of the wire for the 1.2 Ω pod provides less leached metals for Fenton-like chemistry to generate hydroxyl radicals and ROS. However, a mechanistic investigation of ROS formation due to coil resistance was not within the scope of this study.

### 3.2 Effect of Coil Aging on Aerosol Mass and Carbonyl Formation from a Fourth-Generation Pod

We studied pod coils that were aged by usage from new and unused coils up to 5000 puffs. We did not find significant variations, within uncertainty, in measured coil temperature (Figure 4A) nor in aerosol mass produced (Figure 4B) when the coils are new versus aged. However, Figure 4A reveals that the uncertainty in measured coil temperature may be up to ∼25 °C depending on where the thermocouple is seated relative to the coil.

**Figure 5:**
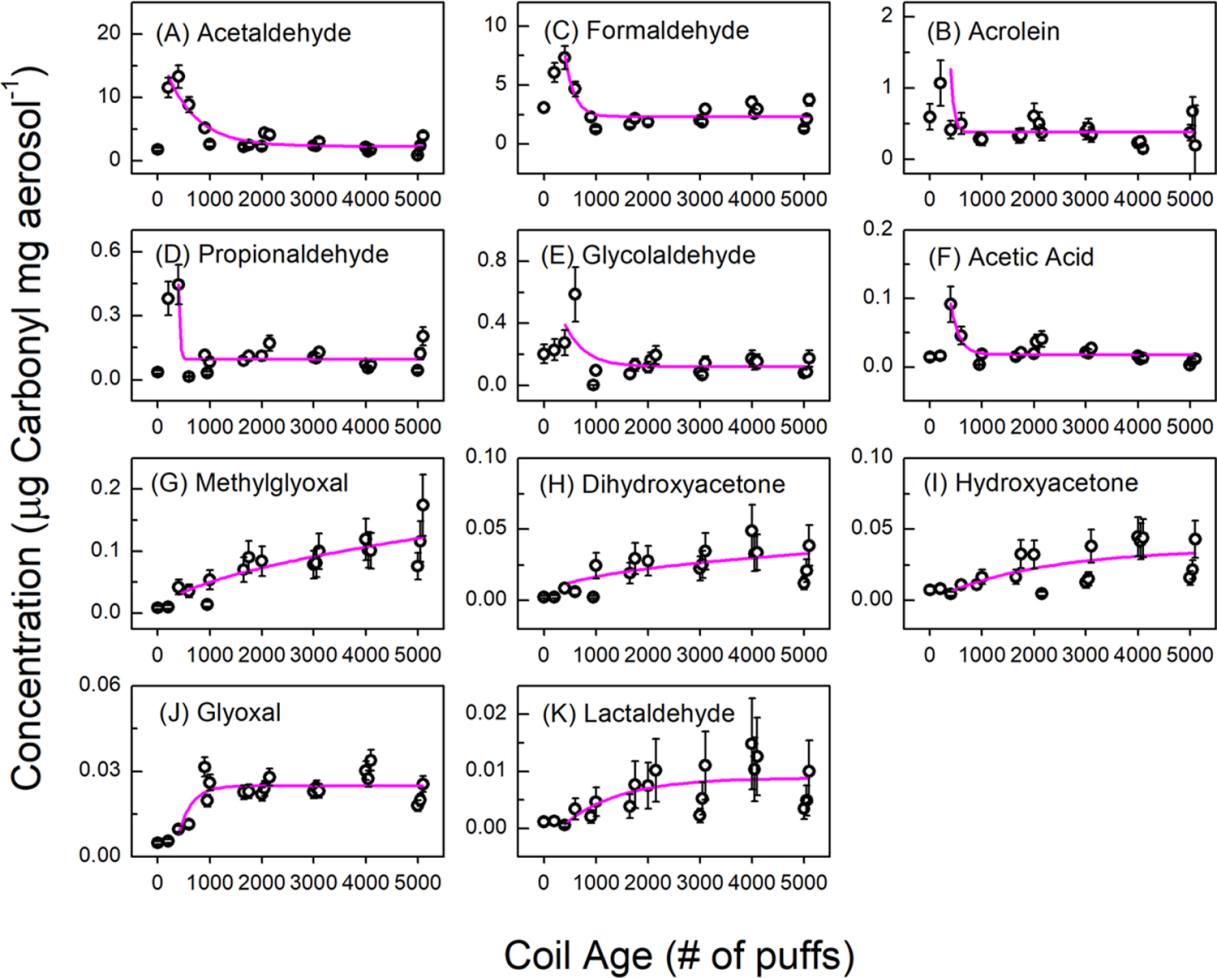
Concentration (μg carbonyl mg aerosol^–1^) of detected carbonyls in the aerosol (average ± SD) as a function of usage-induced coil age using a 1.2 Ω 4th gen pod coil with 2% nicotine salt. Exponential fits are included to guide the eye.

We further evaluated the influence of coil age on aerosol mass-normalized yields of 11 carbonyls, which is presented in Figure 5. We found, consistently with Ureña et al.^38^ and Sleiman et al.,^22^ that acetaldehyde, formaldehyde, and acrolein normalized yields increased when the coil is used for several hundred puffs compared to a new coil. However, the behavior of these three carbonyls in this range of coil aging is neither replicated across all carbonyls nor for the entire range of coil aging in this work. The 4th gen coils we tested reached a maximum yield for acetaldehyde, acrolein, formaldehyde, propionaldehyde, glycolaldehyde, and acetic acid after approximately 500 puffs (Figure 5A–F), and then the yields of these carbonyls drop sharply and reach a stable value after approximately 1000 puffs. Yet, yields of methylglyoxal, dihydroxyacetone, hydroxyacetone, glyoxal, and lactaldehyde were low initially with new coils and increased with coil age (Figure 5G–K). Similarly to coil resistance (Section 3.1), we interpret these diverging trends in carbonyl yields as resulting from the different chemical mechanisms for carbonyl formation.^19^

Saliba et al.^37^ suggests that the surface reactivity of several types of coils increases with a certain age (although the age was not quantified in puffs). While the coils we tested are in a 4th gen device instead of a pyrolysis reactor that was used in that work, the data from the thermal degradation carbonyls suggest that there is indeed an increase in thermal reactivity of the coil (either directly at the surface or in close proximity) up to approximately 500 puffs, which then decreases to baseline again when aged up to 5000 puffs. Upon disassembling used coils that were deemed to have failed, many appeared to have been burned resulting in black discoloration and visibly broken pieces of wire unrelated to the coil inspection. This suggests that the surface quality of the coil changes during aging, which changes its catalytic potential at a nominal temperature in a dynamic way. Potentially, some usage is needed to reach an optimal coil reactivity, which then decreases again as the coil is further used and burned. A study on coil aging using a 3rd gen tank mod showed an increase in formaldehyde and acetaldehyde concentrations (not normalized by aerosol mass) up to 1800 puffs and correlated this carbonyl formation to acute lung injury in mice.^55^ The maximal concentrations of formaldehyde and acetaldehyde in the 3rd gen device required a higher number of puffs than the 4th gen device we tested, which could be due to the different coil size and anatomy, bulk conductance of the 3rd gen apparatus, and variable application of power.

Regarding the carbonyl products that are formed from hydroxyl radical or other ROS (i.e., methylglyoxal, dihydroxyacetone, hydroxyacetone, glyoxal, lactaldehyde), the low yields with new coils that increased to a maximum are likely due to increasing ROS formation as the coil is used and heated.^38^ This is consistent with the increased free radical yield and oral cytotoxicity found by Ureña et al. with increasing coil age in puffs.^38^ As the coil is aged, a secondary source of oxidation can form in the cotton wick soaked with e-liquid, as suggested by Saliba et al.^37^ The cotton also visibly changes from a clean white color to a more yellow-brown color. As the coil degrades over continued usage due to heating, it is possible that metals may leach into the e-liquid as a potential source for ROS, although this remains to be verified.

### 3.3 Aerosol Mass and Carbonyl Formation between a Third-Generation versus a Fourth-Generation Device

Comparisons between a 4th gen and 3rd gen device are subject to a number of variabilities due to the innate differences in device design (e.g., coil shape, surface area, battery supply current, wick capacity, and heating element type); however, this comparison may still provide valuable insight into the differences between “sub-ohm” devices and the lower-powered and newer-generation pods. In this case, we are comparing a stainless steel coil in the 3rd gen tank mod against a kanthal coil in the 4th gen pod. It was found that different coil metal materials (kanthal, stainless steel, nichrome) are responsible for only minor differences in aerosol mass and carbonyl production when normalized by vaping wattage (Figures S7 and S8 and Tables S1–S3). Thus, the differences in metal choice may not be a large source of uncertainty.

**Figure 6:**
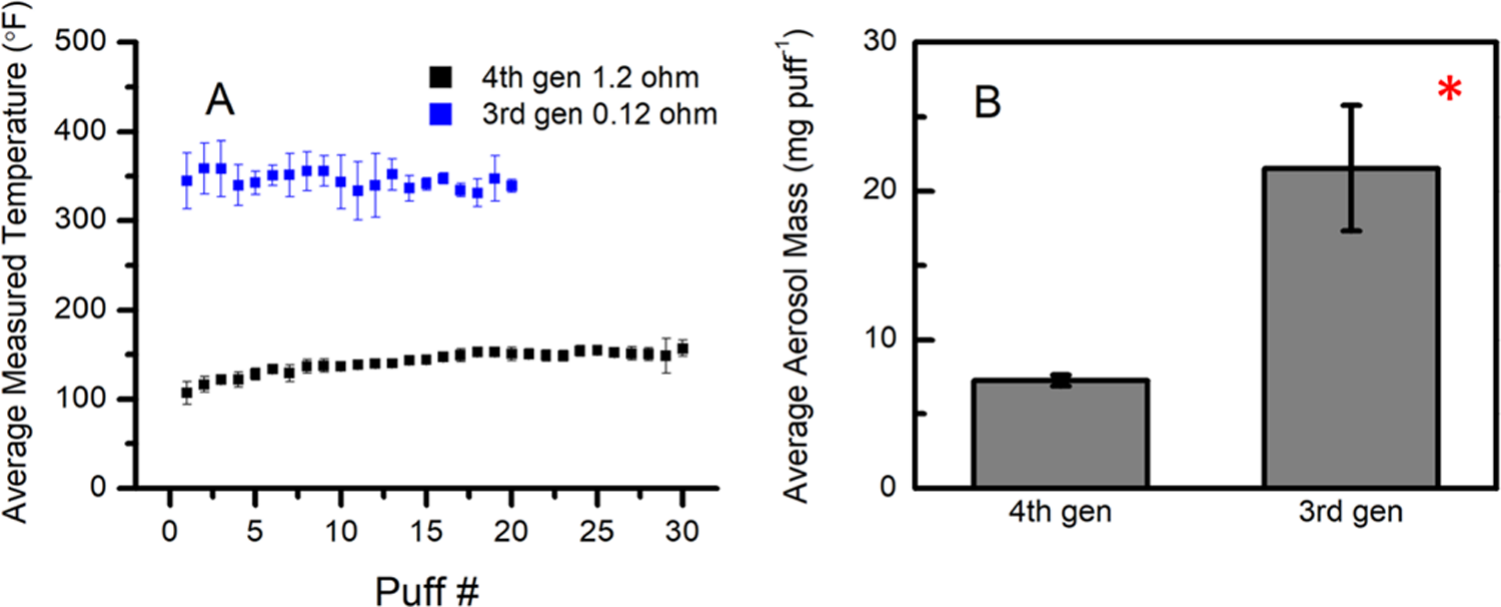
Average aerosol mass (mg puff^–1^) from vaping 2% nicotine salt e-liquid on a 3rd gen tank mod (Freemax stainless steel 0.12 Ω coil) device versus a 4th gen (Vaporesso XROS 1.2 Ω pod) device. An asterisk (*) denotes that the two groups are significantly different (*p* < 0.05). In panel (A), the average temperatures between the 0.12 Ω coil and the 1.2 Ω pod coil were significantly different from each other (*p* < 0.05).

The lowest temperature that the 3rd gen SSF coil can operate to produce aerosol was measured to be 183 °C (361 °F) while the 4th gen device operates at a fixed temperature for each pod resistance (Table 3 and Figure 6A). For the 1.2 Ω coil, the temperature was measured to be 65 °C (149 °F). The higher relative temperature of the 3rd gen coil helps produce a higher aerosol mass (mg puff^–1^) than the lower-temperature 4th gen coil (Table 3 and Figure 6B). These measurements are consistent with the current literature^16,27,31,56^ and with the trends we report from a 4th gen device (Figure 2), where a lower resistance correlates to both a higher measured coil temperature and aerosol mass production.

**Table 3:** Power Output, Measured Temperature, and Aerosol Mass without and with Normalization by Wattage (Average ± SD) Generated from a 4th Gen Device with a 1.2 Ω Vaporesso Pod versus a 3rd Gen Tank Mod Device with a 0.12 Ω Stainless Steel Coil

coil resistance (Ω)	power output (W)	measured temperature	aerosol mass (mg puff^–1^)	aerosol mass (mg puff^–1^ W^–1^)
0.12 (3rd gen)	20.8 ± 1.4	174 ± 11 °C (361 °F)	21.5 ± 4.2	1.03 ± 0.20
1.2 (4th gen)	10^a^	63 ± 8 °C (149 °F)	7.3 ± 0.4	0.73 ± 0.04

a As reported on the manufacturer’s label.

**Figure 7:**
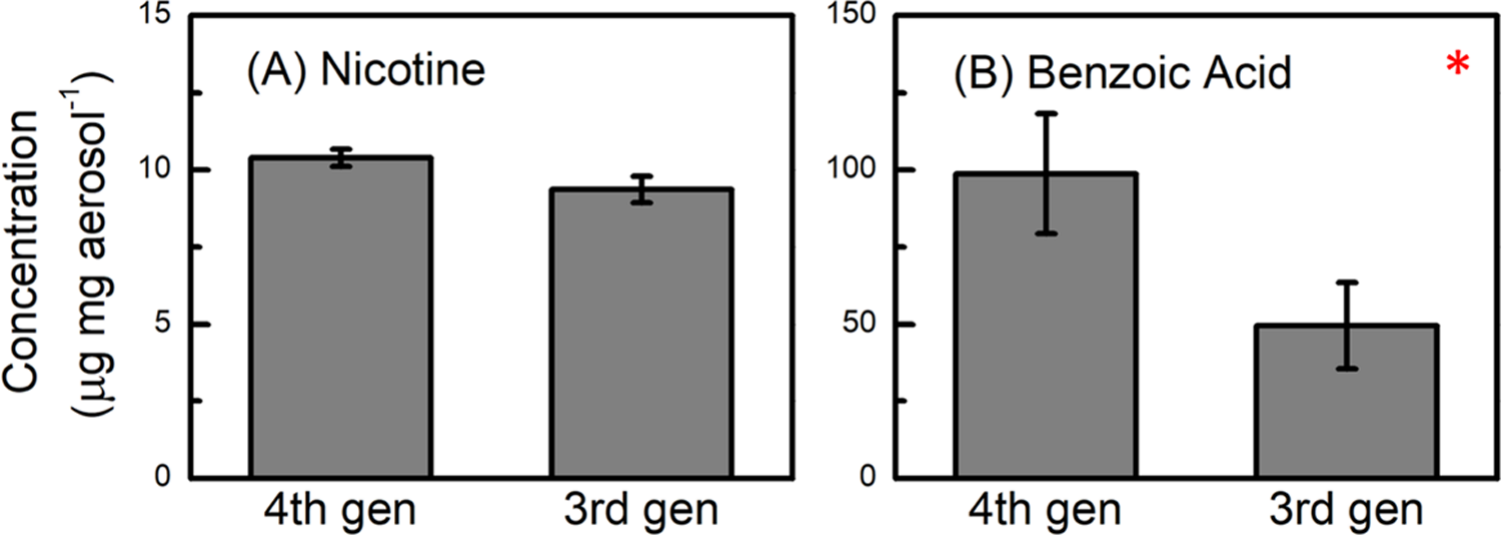
Concentration (μg mg aerosol^–1^) of nicotine and benzoic acid in the aerosol from vaping 2% nicotine salt on a 3rd gen (Freemax stainless steel 0.12 Ω coil) device versus a 4th gen (Vaporesso XROS 1.2 Ω POD) device. Asterisks (*) denote statistically significant differences (*p* < 0.05).

A comparison of aerosolizing the e-liquid between 3rd gen and 4th gen devices confirmed similar nicotine yields in the aerosol (Figure 7A), showing that both devices are aerosolizing the e-liquid similarly. The benzoic acid concentration, however, is lower in the 3rd gen aerosol compared to the 4th gen aerosol. 3rd gen devices are not designed to vape nicotine salts, so it is unclear whether or not the low resistance of the 3rd gen coil does not allow benzoic acid to vaporize as effectively as in a 4th gen device design. The production of carbonyls when vaping freebase nicotine versus nicotine salt in a 3rd gen device is similar for most carbonyls (Figure S9).

**Figure 8:**
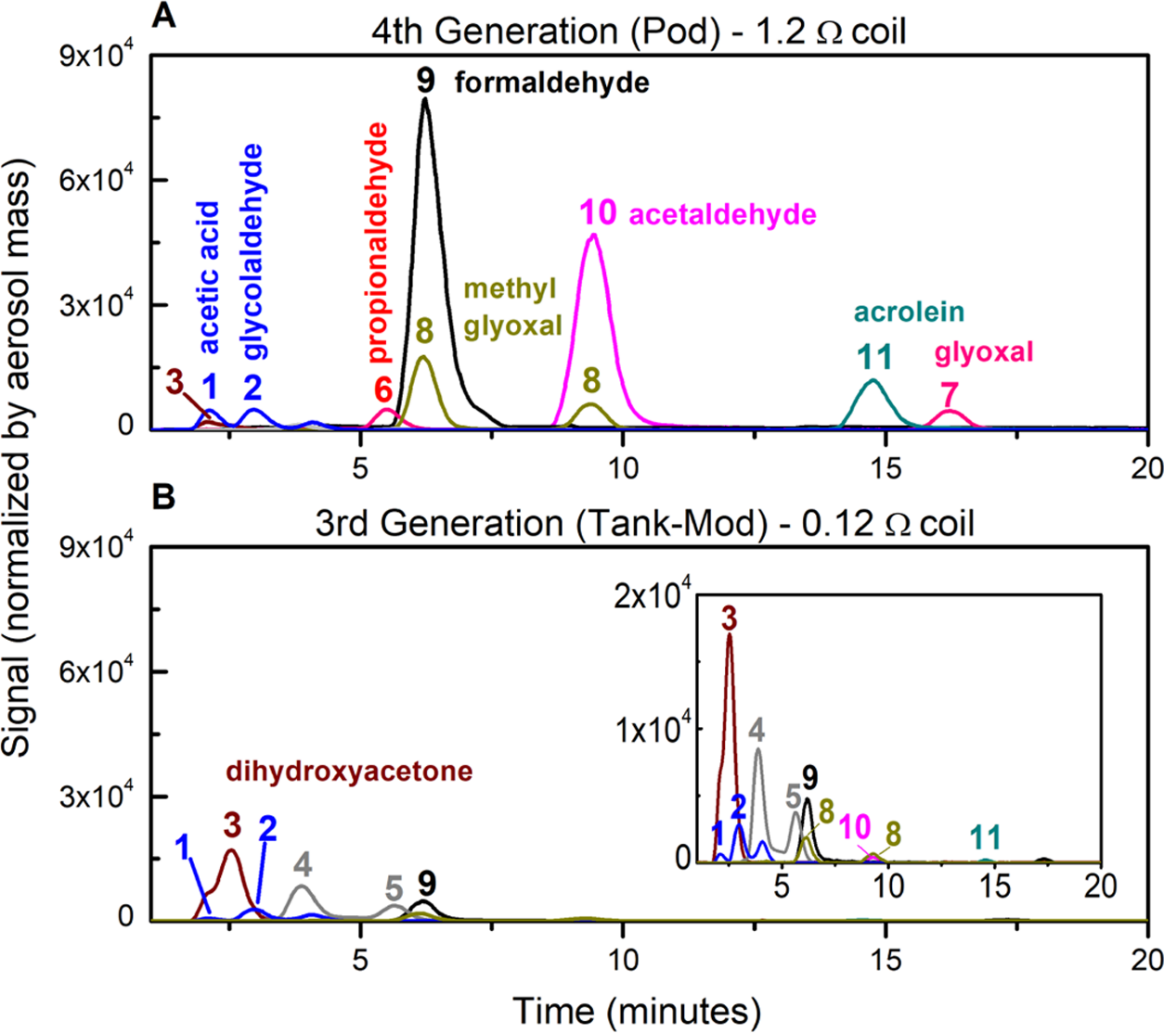
Extracted ion LC-HRMS chromatograms of derivatized carbonyls from a vape aerosol sample produced by a (A) 4th gen device with a 1.2 Ω kanthal coil versus a (B) 3rd gen device with a 0.12 Ω SSF coil using the same 2% nicotine salt e-liquid. Key: (1) acetic acid, (2) glycolaldehyde, (3) dihydroxyacetone, (4) hydroxyacetone, (5) lactaldehyde, (6) propionaldehyde, (7) glyoxal, (8) methylglyoxal, (9) formaldehyde, (10) acetaldehyde, and (11) acrolein. Signals were normalized by the aerosol mass collected on the sampling cartridge, which was kept consistent from both devices.

**Figure 9:**
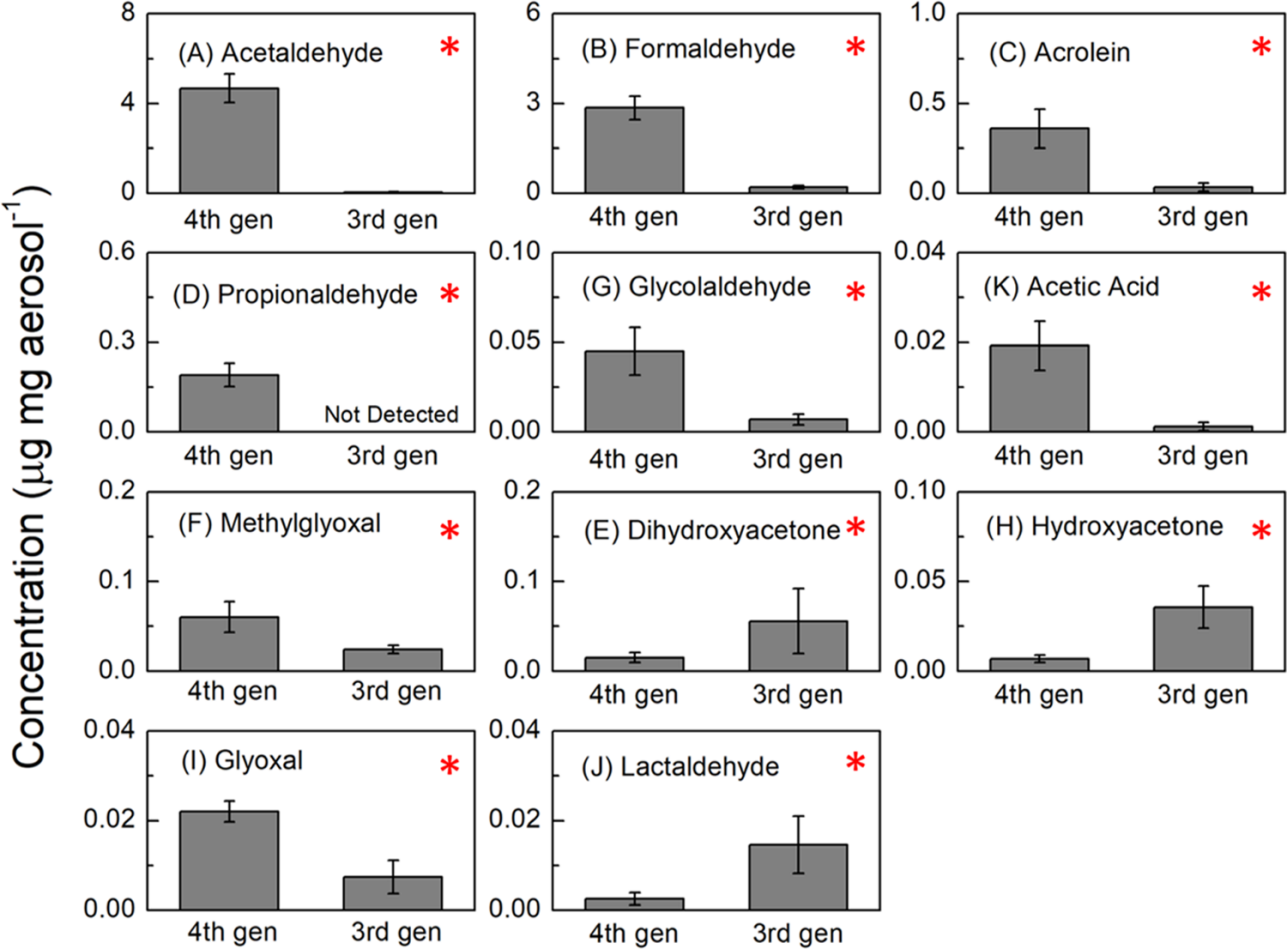
Concentration (μg carbonyl mg aerosol^–1^) of detected carbonyls in the aerosol from vaping 2% nicotine salt on a 3rd gen (Freemax SS316L 0.12 Ω coil) device versus a 4th gen (Vaporesso XROS 1.2 Ω coil) device. Asterisks (*) denote statistically significant differences (*p* < 0.05).

Despite reaching higher temperatures, the concentration yields of most of the carbonyls (μg mg aerosol^–1^) from the sub-ohm 3rd gen device was measured to be significantly lower than the concentration of carbonyls from the 1.2 Ω 4th gen coil (Figures 8 and 9). Total carbonyls and many individual carbonyls (acetaldehyde, formaldehyde, acrolein, propionaldehyde, methylglyoxal, glycolaldehyde, glyoxal, and acetic acid, Figure 9A–D,F,G,I,K) are formed at significantly higher yields in the 4th gen device compared to the 3rd gen. Tabulated results are shown in Table S4. It is worth noting that even when not normalized by aerosol mass (Figure 6B), the 4th gen device produces a higher absolute concentration of carbonyls compared to the 3rd gen device, which was unexpected. Dihydroxyacetone, hydroxyacetone, and lactaldehyde were observed in lower quantities in the 4th gen device (Figure 9E,H,J). These three ROS-derived carbonyls were also found in lower yields at higher resistances (Figure 3) in the 4th gen pods. Overall, the 3rd versus 4th gen device comparisons agree with the coil resistance study in the 4th gen device shown in Figure 3, i.e., lower coil resistance (higher coil temperature) produces lower total carbonyl yields, but the individual trends differ for the thermally derived versus ROS-derived degradation products.

With regards to carbonyl formation by mass fraction, the 3rd gen device produces mostly formaldehyde (∼40%, by carbonyl mass), acetaldehyde (∼10–20%), acrolein (∼5–7%), and hydroxylated carbonyls (of which hydroxyacetone and dihydroxyacetone represents ∼20%) when vaping freebase nicotine (Table S3). In contrast, the 3rd gen device produces much less acetaldehyde and acrolein (5 and 3%, respectively) when vaping nicotine salt (Table S5). The primary carbonyls produced when vaping nicotine salt with a 3rd gen device are formaldehyde (54%) and hydroxylated carbonyls (hydroxyacetone and dihydroxyacetone representing 21%). The 4th gen device produces mostly formaldehyde (35–60%) and acetaldehyde (10–50%) when vaping nicotine salts at various coil resistances, with a much lower representation of acrolein and the hydroxylated carbonyls (Table S5).

## 4 Conclusions

Coil resistance has a strong and predictable inverse relationship with coil temperature, which directly drives aerosol formation. However, carbonyl formation as a function of coil resistance (i.e., temperature) is complex. Carbonyls derived primarily from thermal degradation mechanisms of PG and VG (e.g., formaldehyde, acetaldehyde, acrolein, propionaldehyde, etc.) tend to be formed at higher concentration yields with higher coil resistances (and lower coil temperatures). This may be due to higher dissipated temperatures in the e-liquid at the higher voltage drop. However, such a hypothesis remains to be tested. Carbonyls that are derived primarily from ROS-derived mechanisms (e.g., hydroxyacetone, dihydroxyacetone, methylglyoxal, glycolaldehyde, lactaldehyde, etc.) may be formed in higher yields at lower resistances. In general, coil age does not significantly affect coil temperature and aerosol formation but has a large impact on carbonyl yields. It appears that large-scale processes like aerosolization and wire heating were unaffected by the changes in coil resistivity and metal surface characteristics as a coil ages; however, small-scale processes like chemical transformations are highly affected. Thermal degradation carbonyls, again, show different trends with coil age (0–5000 puffs) compared to ROS-derived carbonyls. The thermal degradation carbonyl yields rise sharply to a maximum at approximately 500 puffs in the 4th gen device and then decline to a baseline. In contrast, ROS-derived carbonyls show a rise to a maximum trend throughout. We also find that, despite innate differences in device design, the 3rd gen versus 4th gen comparison of aerosol formation and carbonyl yields mirrors the trends that are controlled by coil resistance. There is not a significant difference in nicotine yield between 3rd and 4th gen devices.

This work is supportive of the conclusions of Beauval et al.^33^ and Talih et al.^34^ that the “lower-powered” devices such as the 4th gen pods meant to vape nicotine salts do not necessarily produce less carbonyl toxicants when normalized with aerosol mass. The results are more nuanced and require consideration of the chemical mechanisms of carbonyl formation, as well as the specific resistances and construction (Figures S1 and S2) of each coil under study. This work is also consistent with the results of Pinkston et al.;^15^ the device design or coil resistance of the 4th gen may play a role in the toxicity of the aerosol along with the use of the nicotine salt.

While this work offers a number of new insights, we also note certain limitations. It is important to emphasize that the results and conclusions of this work should be considered with regards to the experimental conditions tested. Vaping of a nicotine salt e-liquid was performed on a 3rd gen device in Section 3.3 for experimental consistency, which is not the typical device used by most users for nicotine salts (although the combination of nicotine salts and sub-ohm devices does occur in some use scenarios, and new “sub-ohm nicotine salts” are being introduced to the commercial market). However, this may only have minor effects on the experimental design, as the addition of the acid did not notably affect carbonyl yields (Figure S9). We also used a different PG/VG ratio (30:70) than the typical ratio for many nicotine salts on the commercial market (50:50). We opted to be consistent with the JUUL patent that uses a 30:70 PG/VG ratio. The discrepancies resulting from the carrier solvent ratio may also be minor, as Li et al. has shown the that carbonyl yields were similar between a 50:50 PG/VG and 30:70 PG/VG solutions.^19^ Limitations to this work also include the fact that flavored e-liquids were not studied, which could impact the concentration of carbonyls produced from these devices. In addition, higher nicotine salt content (∼5%) can be used in fourth-generation devices and may also impact the generation of aerosol mass, user puffing patterns, and exposure to carbonyls. Higher nicotine aerosol concentrations may influence users to alter puffing patterns and frequency to achieve a targeted nicotine dose (e.g., nicotine titration), which in turn would influence user exposures to carbonyls and other aerosol constituents. Another limitation may be the differences in the sampling flow rate between 4th gen and 3rd gen devices, even when the puff volume is kept consistent within uncertainty. It is likely the effects of such discrepancies can be accounted for by normalizing the collected data by aerosol mass. Disposable vaping devices were not studied in this work because they would be difficult to study in a controlled manner (e.g., varying e-liquid formulation, device components, device performance) and would challenge our ability to extract fundamental information regarding carbonyl formation; however, they should be tested in future studies due to their high consumer popularity.

Certain implications for health-related studies are gained from this work. First, it may be beneficial to adopt a definition of coil age based on usage as there does not appear to be a dichotomy of “aged” versus new coil for the production of different carbonyls across device types. For some of the most abundant toxic carbonyls, there is a maximum activity level of the 4th gen coil in the middle range of the coil’s lifetime that decreases again with further aging. There is currently no practical consensus on when to change out pods in the 4th gen Vaporesso device or others. However, a popular online vaping magazine suggests to replace with a new Vaporesso XROS pod “when the coil dies,”^57^ which did not occur even after 5000 puffs in our study. Thus, the e-cigarette user may be exposed to a highly dynamic aerosol carbonyl composition in the coil’s lifetime. The increase in ROS-initiated carbonyls over the coil’s lifetime is consistent with in vitro and in vivo toxicity studies with aged coils.^38,55^ This study suggests that ROS formation and cytotoxicity endpoints (more toxic at ≥2000 puffs in our study) may be different from health endpoints related only to the exposure of toxic carbonyls (more toxic at ∼500 puffs in our study). Although this work focuses on carbonyls, the toxicity of e-cigarette aerosols is not solely driven by carbonyls. A similar analysis for metals, free radicals, and other toxic or potentially toxic compounds due to coil aging may be informative for health risk assessment. For researchers performing exposure and toxicity assessments on e-cigarettes, differently aged coils may cause discrepancies and inconsistencies in data. Thus, researchers should be cognizant of the age of the coils being used in experiments.

Furthermore, researchers and e-cigarette users should not assume that the higher-powered sub-ohm devices (or lower-resistance coils in general) are less safe with regards to carbonyl formation yields because they produce more aerosols. The lower-powered higher-resistance coils may produce more total carbonyls per mg aerosol (and thus, nicotine) that is inhaled in our study using unflavored e-liquids. Users that self-titrate nicotine usage may be exposed to higher amounts of certain carbonyls at the same inhaled aerosol mass with the lower-powered 4th gen products under the conditions tested in this study. As a final point, researchers should be careful to not compare data across different devices, coil age, and coil anatomy without accounting for the aerosol chemistry outcomes that these parameters may control or for the specific formation mechanisms of the carbonyl emission that may alter their trends with each parameter.

## Supporting Information Available

Device deconstruction photos for the 3rd and 4th gen devices; aerosol mass produced by the 3rd gen device when airway valve is open versus closed; HPLC-HRMS calibration for carbonyls; aerosol mass captured by the quartz filter for 3rd and 4th gen device sampling; gas chromatography-MS (GC-MS) calibration for nicotine; aerosol mass from different 3rd gen coils; carbonyl concentrations from different 3rd gen coils normalized by mass and wattage; carbonyl concentrations from vaping freebase nicotine and nicotine salt on a 3rd gen device; table of variables in 3rd gen coil material experiments; table reporting aerosol mass and temperature from 3rd gen coils of different metal materials, without and with normalization by wattage; table reporting nicotine, benzoic acid, and carbonyl concentrations for all nicotine salt experiments (PDF)
