# Supplementary material for: Carbonyls and Aerosol Mass Generation from Vaping Nicotine Salt Solutions Using Fourth- and Third-Generation E-Cigarette Devices: Effects of Coil Resistance, Coil Age, and Coil Metal Material
Source: Chem Res Toxicol. 2023 Sep 12;36(10):1599–610. doi: 10.1021/acs.chemrestox.3c00172 (PMC10583227; doi:10.1021/acs.chemrestox.3c00172)
Supplement: Supplementary file 1 — tx3c00172_si_001.pdf [file tx3c00172_si_001.pdf]

Supporting Information

Carbonyls and aerosol mass generation from vaping nicotine salt solutions using fourth- and third-generation e-cigarette devices: Effects of coil resistance, coil age, and coil metal material

Lillian N. Tran,[1] Elizabeth Y. Chiu,[1] Haylee C. Hunsaker,[2] Kuan-chen Wu,[2] Brett A. Poulin,[1] Amy K. Madl,[3] Kent E. Pinkerton,[3] Tran B. Nguyen[1*]

1. Department of Environmental Toxicology, University of California, Davis, Davis CA 95616, USA
2. Department of Chemistry, University of California, Davis, Davis CA 95616, USA
3. Center for Health and the Environment, University of California Davis, Davis, CA 95616

**author to whom correspondence should be directed: T.B. Nguyen (tbn@ucdavis.edu)*

Table of Contents

Picture of deconstructed Vaporesso POD coils S2
Picture of deconstructed 3rd gen coils S2
Aerosol mass produced by the 3rd gen device when airway valve is open versus closed S3
HPLC-HRMS concentration calibration curves of carbonyl DNPH-hydrazones S3
Aerosol mass captured by the quartz filter for 3rd and 4th gen device sampling S4
GC-MS concentration calibration curve of nicotine S4
Aerosol mass data from different 3rd gen coils S5
Carbonyl concentrations from different 3rd gen coils normalized by mass and wattage S5
Carbonyl concentrations from vaping freebase nicotine and nicotine salt on a 3rd gen device S6
Table of variables in 3rd gen coil material experiments S7
Table reporting aerosol mass temperature from 3rd gen coils of different metal materials S7
Table reporting nicotine, benzoic acid, and carbonyl concentrations from different 3rd gen coils (normalized by mass) S8
Table reporting nicotine, benzoic acid, and carbonyl concentrations from different 3rd gen coils (normalized by mass and wattage) S9
Table reporting nicotine, benzoic acid, and carbonyl concentrations of the 3rd gen SSF coil and the 4th gen 1.2, 0.8, and 0.6 ohm pod coils. S10

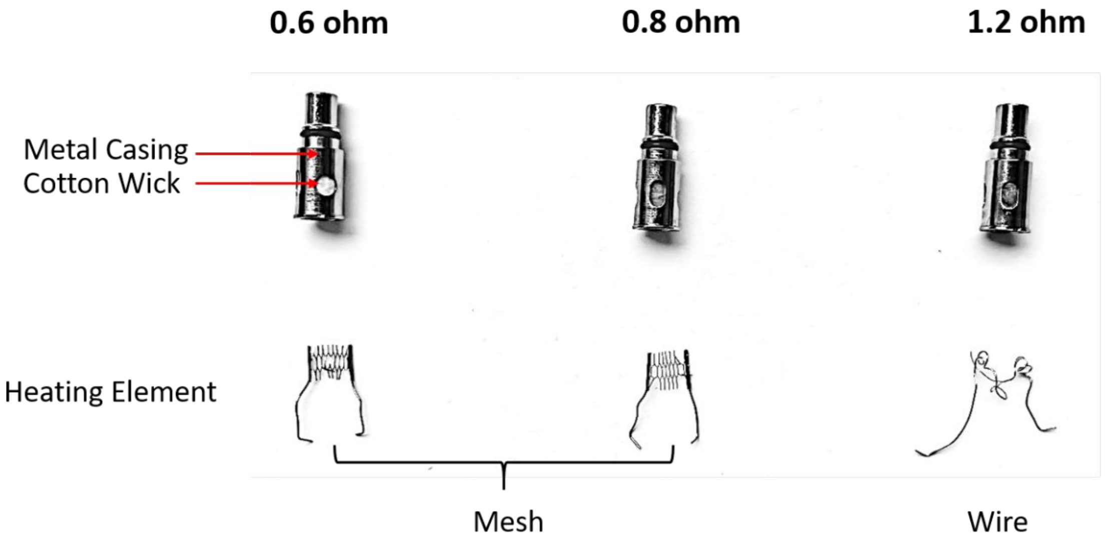

Figure S1. Deconstructed coils in the Vaporesso XROS 4th gen pod device at different coil resistances.

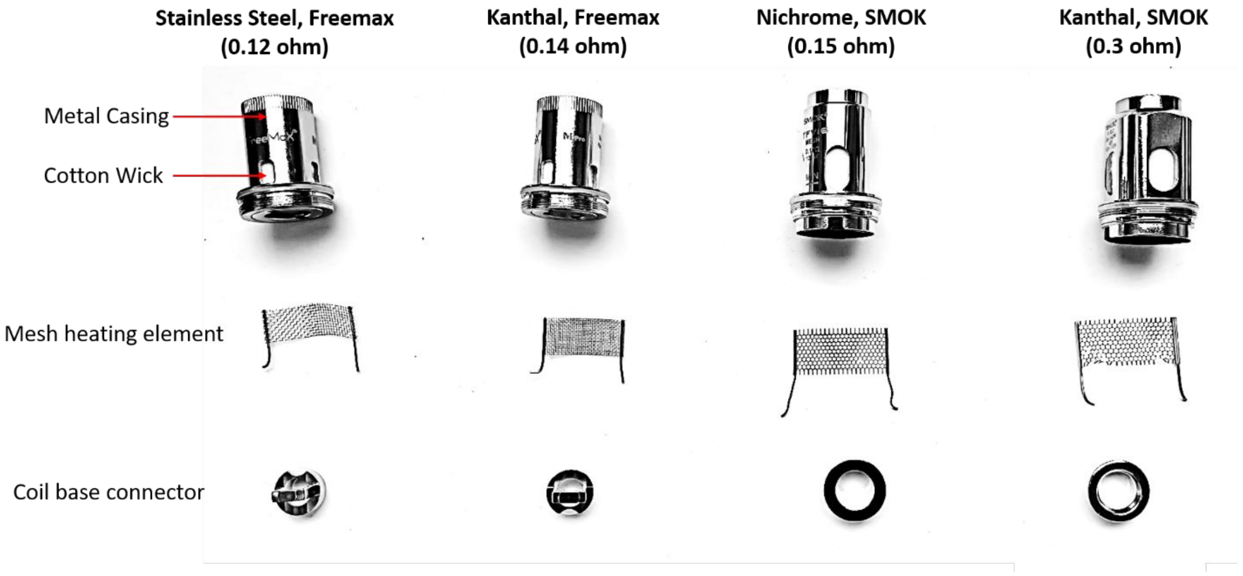

Figure S2. Deconstructed coils in the Lost Vape 3rd gen tank mod device for different coil construction material and resistances.

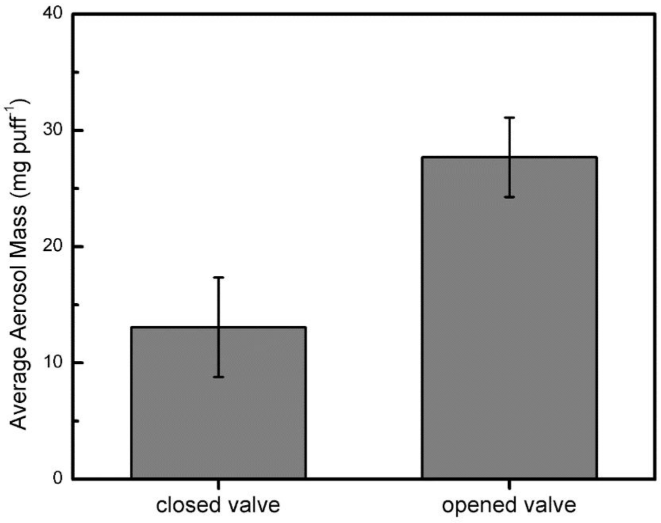

Figure S3. Aerosol mass (mg puff^{-1}) produced by a 3rd gen device with the airflow valve open versus closed.

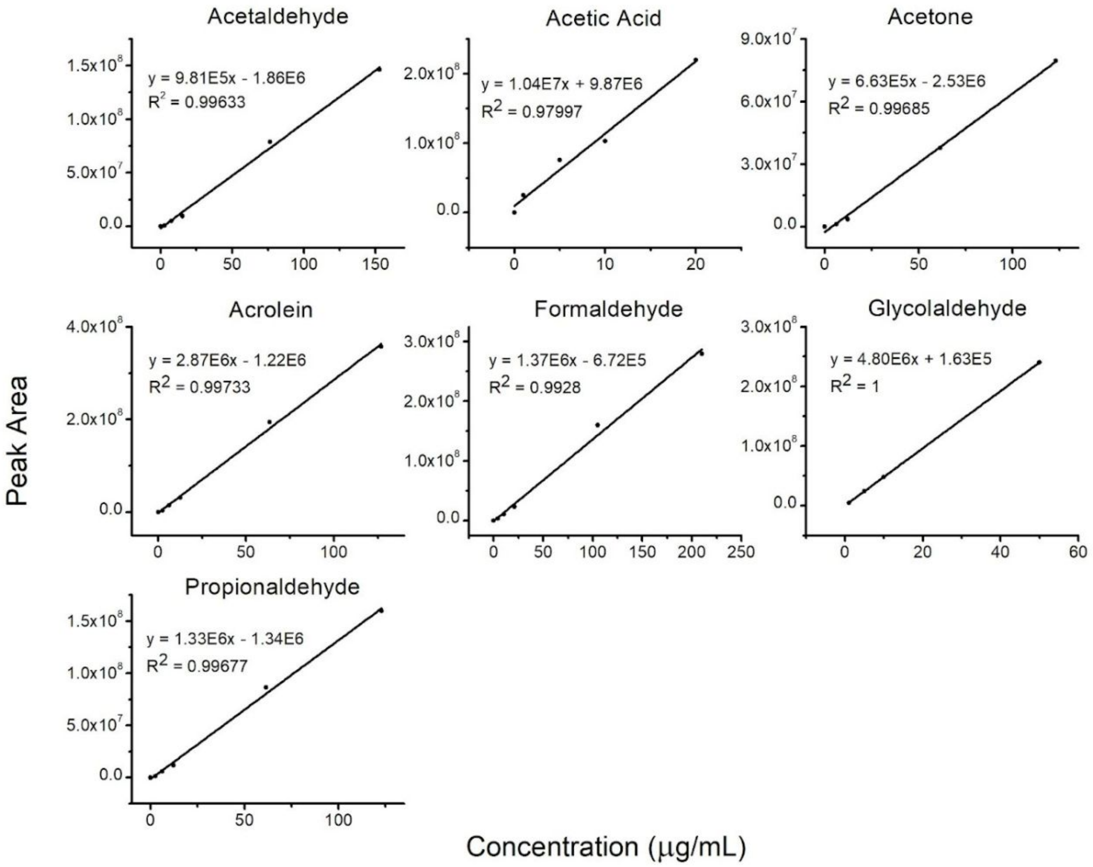

Figure S4. HPLC-HRMS calibration curves for seven carbonyl-DNPH hydrazones: Acetaldehyde-, Acetic Acid-, Acetone-, Acrolein-, Formaldehyde-, Glycolaldehyde-, Propionaldehyde-DNPH.

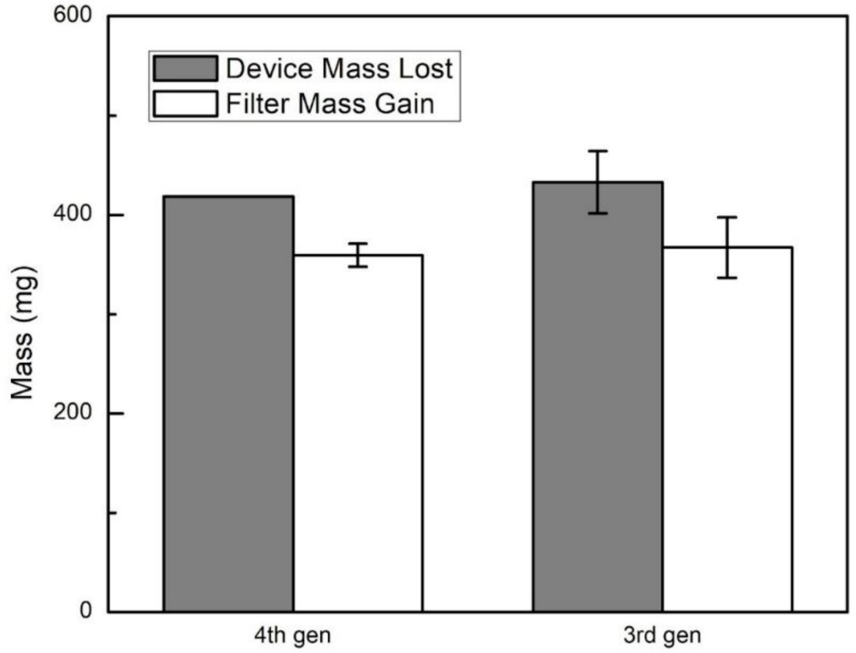

Figure S5. Comparison of aerosol mass collected on the filter versus the total mass loss of the device.

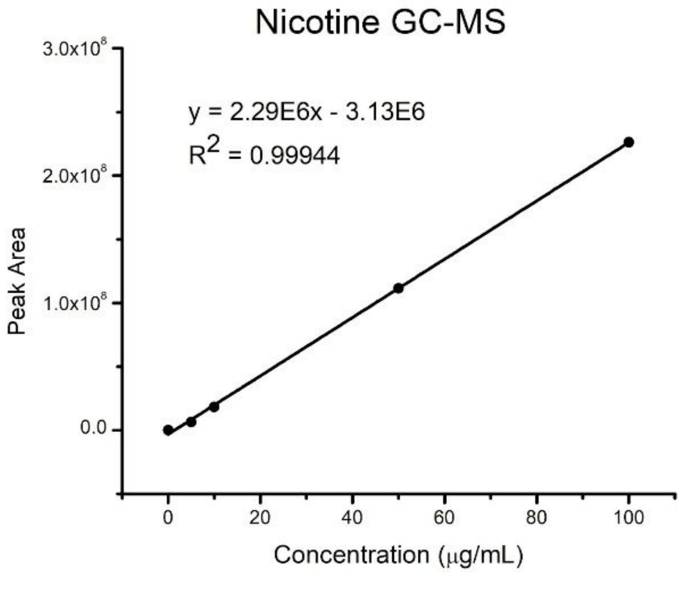

Figure S6. GC-MS calibration curve for nicotine.

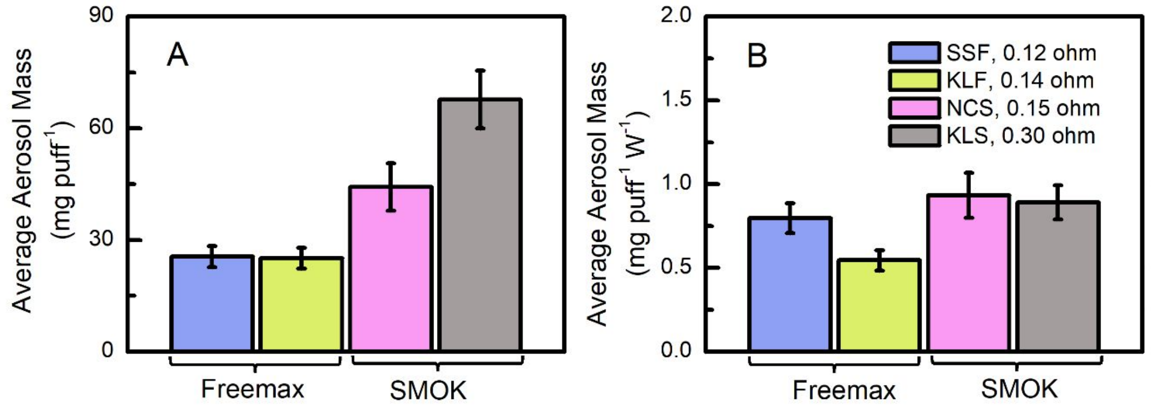

Figure S7. Aerosol mass (mg puff^{-1}) (A) and normalized by power (mg puff $^{-1}$ W^{-1}) (B) (average ± SD) generated from vaping 0.6% freebase nicotine on different 3rd generation coils.

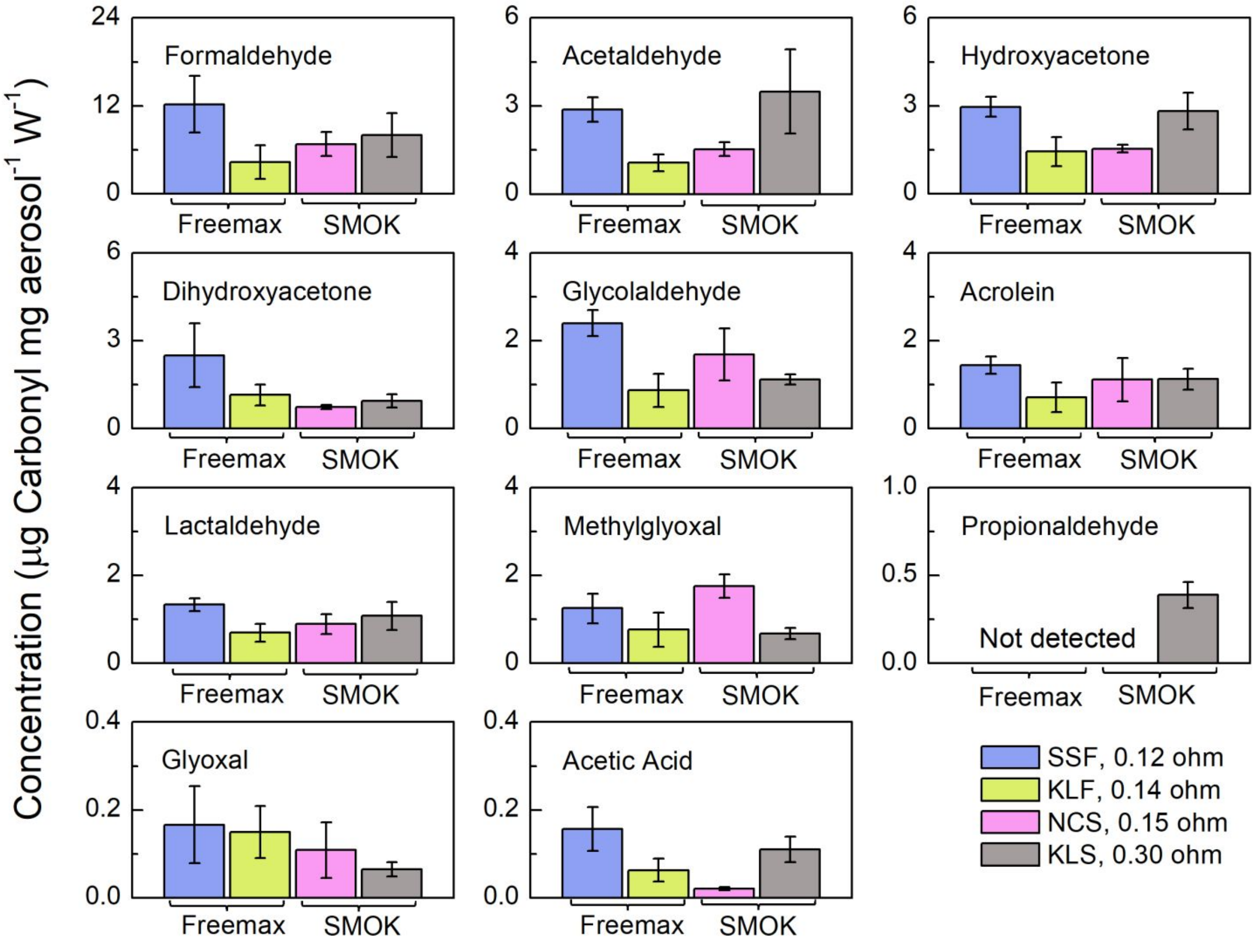

Figure S8. Concentration of carbonyls (µg carbonyl mg aerosol^{-1} W^{-1}) from vaping 0.6% freebase nicotine on different 3rd gen coils.

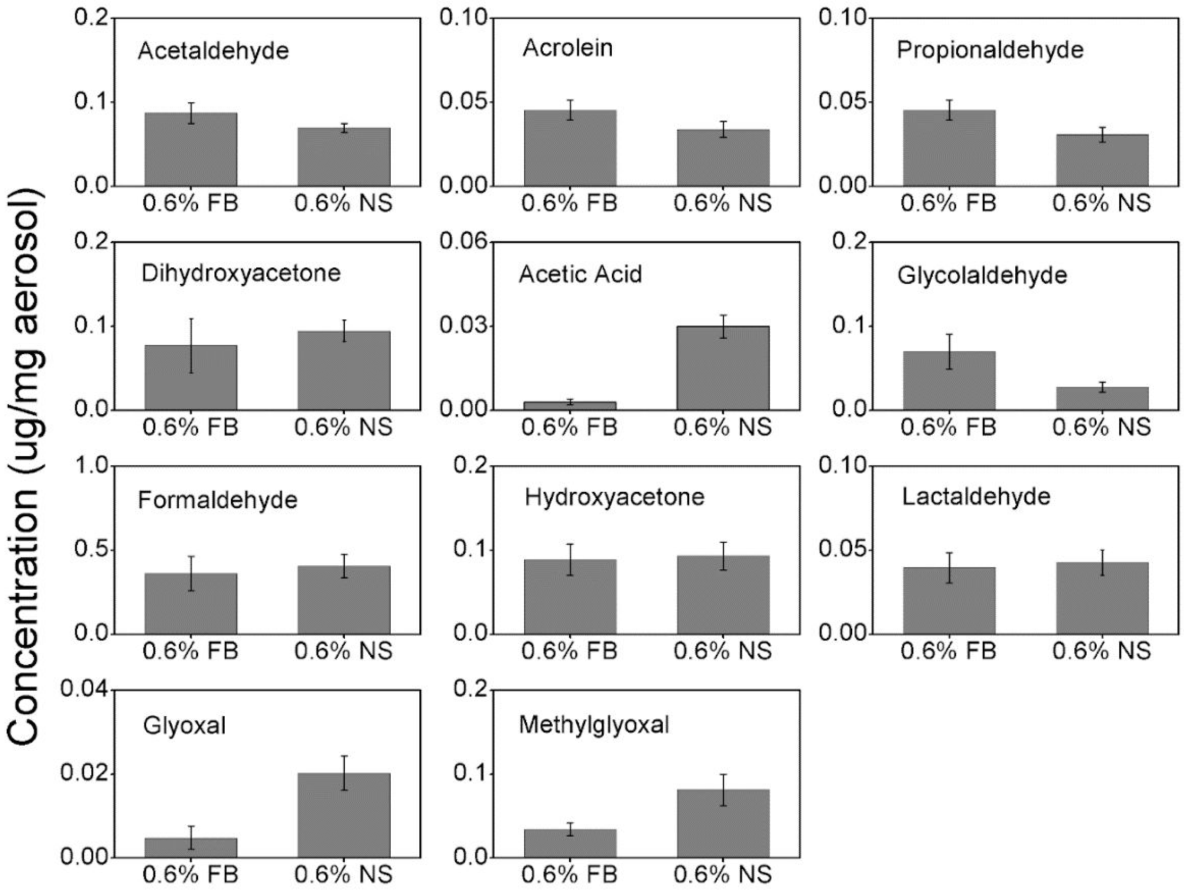

Figure S9. Concentration of carbonyls (μg carbonyl mg aerosol^{-1}) from vaping 0.6% freebase nicotine versus 0.6% nicotine salt (1:1 nicotine:benzoic acid) on a SSF coil in the 3rd gen device.

Table S1. Experiment conditions for the study of coil metal construction material using the 3rd gen tank mod device. All studies of the coil metal material were performed using 0.6% freebase nicotine.

**Device type**	**Coil Material**	**Brand**	**Coil Resistance (ohm)**
3rd gen	Stainless Steel (SSF)	Freemax	0.12
3rd gen	Kanthal (KLF)	Freemax	0.14
3rd gen	Kanthal (KLS)	SMOK	0.3
3rd gen	Nichrome (NCS)	SMOK	0.15

Table S2. Power output, temperature, and aerosol mass without and with normalization by wattage (average ± SD) from vaping 0.6% freebase nicotine on different 3rd gen coils.

	Power output to achieve 204-232 ºC (W)	Measured Temperature	Aerosol Mass (mg $puff^{-1}$)	Aerosol Mass (mg $puff^{-1}$ W^{-1})
Stainless Steel (Freemax)	32.4 ± 1.8	229 ± 4 ºC (444 ºF)	25.56 ± 2.87	0.80 ± 0.09
Kanthal (Freemax)	46.1 ± 2.6	234 ± 4 ºC (453 ºF)	25.14 ± 2.80	0.54 ± 0.06
Nichrome (SMOK)	47.4 ± 4.9	234 ± 9 ºC (453 ºF)	44.21 ± 6.37	0.93 ± 0.13
Kanthal (SMOK)	76.1 ± 19.2	236 ± 2 ºC (456 ºF)	66.03 ± 19.92	0.80 ± 0.22

Table S3. Average concentrations of carbonyls (μg carbonyl mg aerosol^{-1}) ± SD in the aerosol from vaping 0.6% freebase nicotine on different 3rd generation coils. Carbonyl mass fractions (normalized by total carbonyl mass) are reported in parentheses.

Coil Material	Form-aldehyde	Acet-aldehyde	Acrolein	Propion-aldehyde	Dihydroxy-acetone	Acetic Acid	Glycol-aldehyde	Hydroxy-acetone	Lact-aldehyde	Methyl-glyoxal	Glyoxal
SSF (0.12 ohm)	0.379 ± 0.104 (0.444)	0.087 ± 0.012 (0.101)	0.046 ± 0.006 (0.053)	Not detected (0)	0.080 ± 0.035 (0.094)	0.004 ± 0.002 (0.005)	0.077 ± 0.009 (0.089)	0.095 ± 0.009 (0.110)	0.043 ± 0.005 (0.050)	0.040 ± 0.011 (0.047)	0.005 ± 0.003 (0.006)
KLF (0.14 ohm)	0.198 ± 0.105 (0.378)	0.049 ± 0.013 (0.094)	0.033 ± 0.015 (0.063)	Not detected (0)	0.053 ± 0.017 (0.102)	0.003 ± 0.001 (0.006)	0.04 ± 0.017 (0.077)	0.066 ± 0.023 (0.126)	0.032 ± 0.009 (0.061)	0.037 ± 0.009 (0.080)	0.007 ± 0.003 (0.013)
KLS (0.3 ohm)	0.610 ± 0.230 (0.431)	0.265 ± 0.109 (0.189)	0.085 ± 0.037 (0.060)	0.029 ± 0.006 (0.021)	0.057 ± 0.005 (0.040)	0.002 ± 2.80E-4 (0.001)	0.128 ± 0.045 (0.092)	0.117 ± 0.010 (0.082)	0.068 ± 0.017 (0.048)	0.041 ± 0.009 (0.031)	0.008 ± 0.005 (0.006)
NCS (0.15 ohm)	0.321 ± 0.077 (0.392)	0.072 ± 0.011 (0.089)	0.054 ± 0.011 (0.065)	Not detected (0)	0.045 ± 0.011 (0.055)	0.005 ± 0.001 (0.007)	0.053 ± 0.006 (0.065)	0.133 ± 0.029 (0.162)	0.051 ± 0.015 (0.062)	0.083 ± 0.011 (0.099)	0.003 ± 0.001 (0.004)

Table S4. Average concentrations of carbonyls normalized by power (μg carbonyl mg aerosol^{-1} W^{-1}) ± SD in the aerosol from vaping 0.6% freebase nicotine on different 3rd generation coils.

Coil Material	Form-aldehyde	Acet-aldehyde	Acrolein	Propion-aldehyde	Dihydroxy-acetone	Acetic Acid	Glycol-aldehyde	Hydroxy-acetone	Lact-aldehyde	Methyl-glyoxal	Glyoxal
SSF (0.12 ohm)	12.19 ± 3.87	2.87 ± 0.42	1.44 ± 0.19	Not Detected	2.49 ± 1.09	0.16 ± 0.05	2.40 ± 0.30	2.97 ± 0.33	1.34 ± 0.15	1.25 ± 0.34	0.17 ± 0.09
KLF (0.14 ohm)	4.31 ± 2.28	1.07 ± 0.29	0.71 ± 0.34	Not Detected	1.15 ± 0.36	0.06 ± 0.03	0.88 ± 0.37	1.44 ± 0.50	0.69 ± 0.20	0.77 ± 0.39	0.15 ± 0.06
KLS (0.3 ohm)	8.01 ± 3.03	3.49 ± 1.43	1.12 ± 0.49	0.39 ± 0.07	0.74 ± 0.07	0.02 ± 3.00E-3	1.69 ± 0.59	1.54 ± 0.13	0.89 ± 0.23	0.67 ± 0.12	0.11 ± 0.06
NCS (0.15 ohm)	6.78 ± 1.63	1.53 ± 0.24	1.13 ± 0.24	Not Detected	0.95 ± 0.23	0.11 ± 0.03	1.12 ± 0.12	2.82 ± 0.62	1.08 ± 0.32	1.76 ± 0.26	0.07 ± 0.02

Table S5. Average concentration yields of carbonyls, nicotine, and benzoic acid (all in µg mg aerosol^{-1}) ± SD from vaping 2% nicotine salt on different coil resistances in the 4th generation Vaporesso pods compared to the 3rd generation SS316 coil. Not applicable (N/A) means the sample was not collected. Carbonyl mass fractions (normalized by total carbonyl mass) are reported in parentheses.

Coil resistance and device	Nic-otine	Benzoic Acid	Form-aldehyde	Acet-aldehyde	Acrolein	Propion-aldehyde	Dihydroxy-acetone	Acetic Acid	Glycol-aldehyde	Hydroxy-acetone	Lact-aldehyde	Methyl-glyoxal	Glyoxal
SS316 (0.12 ohm) tank mod	9.36 ± 0.42	49.52 ± 14.00	0.20 ± 0.05<br>(0.46)	0.05 ± 0.01<br>(0.12)	0.03 ± 0.02<br>(0.07)	Not Detected<br>(0)	0.06 ± 0.04<br>(0.14)	1.2E-3 ± 9.4E-4<br>(2.7-3)	6.9E-3 ± 2.9E-3<br>(0.02)	0.04 ± 0.01<br>(0.09)	1.5E-2± 6.3E-3<br>(0.03)	2.4E-2 ± 4.8E-3<br>(0.06)	7.5E-3 ± 3.7E-3<br>(0.02)
1.2 ohm pod	10.38 ± 0.28	98.66 ± 19.49	2.85 ± 0.38<br>(0.35)	4.68 ± 0.64<br>(0.57)	0.36 ± 0.11<br>(0.04)	0.19 ± 0.04<br>(0.02)	1.5E-2 ± 5.6E-3<br>(1.8E-3)	1.9E-2 ± 5.5E-2<br>(2.4E-3)	4.5E-2 ± 1.3E-2<br>(5.5E-3)	6.9E-3 ± 2.0E-3<br>(8.4E-4)	2.6E-3 ± 1.4E-3<br>(3.3E-4)	6.0E-2 ± 1.7E-2<br>(7.3E-3)	2.2E-2 ± 2.4E-3<br>(2.7E-3)
0.8 ohm pod	N/A	106.90 ± 0.79	2.34 ± 0.17<br>(0.52)	1.36 ± 0.15<br>(0.28)	0.28 ± 0.06<br>(0.05)	0.10 ± 5.0E-3<br>(0.02)	5.4E-2 ± 7.5E-3<br>(0.01)	9.6E-3 ± 6.2E-3<br>(2.0E-3)	0.23 ± 0.04<br>(0.06)	8.3E-2 ± 2.0E-2<br>(0.02)	2.9 ± 7.2E-3<br>(7.0E-3)	1.04E-1 ± 9.5E-3<br>(0.02)	1.9E-2 ± 1.0E-2<br>(3.5E-3)
0.6 ohm pod	N/A	93.35 ± 27.42	0.85 ± 0.10<br>(0.57)	0.17 ± 0.01<br>(0.11)	4.7E-2 ± 1.0E-2<br>(0.03)	3.19E-2 ± 2.1E-2<br>(0.02)	9.0E-2± 8.2E-3<br>(0.06)	1.2E-2 ± 6.9E-3<br>(8.1E-3)	1.6E-2 ± 6.8E-3<br>(0.1)	5.2E-2 ± 2.3E-3<br>(0.03)	2.4E-2 ± 3.1E-3<br>(0.02)	4.3E-2 ± 4.3E-3<br>(0.03)	2.9E-2 ± 3.6E-3<br>(0.02)
